# Gut Microbiota-Derived Short-Chain Fatty Acids: Novel Regulators of Intestinal Serotonin Transporter

**DOI:** 10.3390/life13051085

**Published:** 2023-04-26

**Authors:** Berta Buey, Ana Forcén, Laura Grasa, Elena Layunta, Jose Emilio Mesonero, Eva Latorre

**Affiliations:** 1 Departamento de Farmacología, Fisiología y Medicina Legal y Forense, Facultad de Veterinaria, Universidad de Zaragoza, 50013 Zaragoza, Spain; 2 Departamento de Bioquímica y Biología Molecular y Celular, Facultad de Ciencias, Universidad de Zaragoza, 50009 Zaragoza, Spain; 3 Instituto de Investigación Sanitaria de Aragón (IIS Aragón), 50009 Zaragoza, Spain; 4 Instituto Agroalimentario de Aragón-IA2, Universidad de Zaragoza-CITA, 50013 Zaragoza, Spain

**Keywords:** serotonin, immunity, intestine, microbiota-gut–brain axis, SERT

## Abstract

Serotonin (5-HT) is a key neurotransmitter synthesized both in the gut and the central nervous system. It exerts its signaling through specific receptors (5-HTR), which regulate numerous behaviors and functions such as mood, cognitive function, platelet aggregation, gastrointestinal motility, and inflammation. Serotonin activity is determined mainly by the extracellular availability of 5-HT, which is controlled by the serotonin transporter (SERT). Recent studies indicate that, by activation of innate immunity receptors, gut microbiota can modulate serotonergic signaling by SERT modulation. As part of its function, gut microbiota metabolize nutrients from diet to produce different by-products, including short-chain fatty acids (SCFAs): propionate, acetate, and butyrate. However, it is not known whether these SCFAs regulate the serotonergic system. The objective of this study was to analyze the effect of SCFAs on the gastrointestinal serotonergic system using the Caco-2/TC7 cell line that expresses SERT and several receptors constitutively. Cells were treated with different SCFAs concentrations, and SERT function and expression were evaluated. In addition, the expression of 5-HT receptors 1A, 2A, 2B, 3A, 4, and 7 was also studied. Our results show that the microbiota-derived SCFAs regulate intestinal serotonergic system, both individually and in combination, modulating the function and expression of SERT and the 5-HT1A, 5-HT2B, and 5-HT7 receptors expression. Our data highlight the role of gut microbiota in the modulation of intestinal homeostasis and suggest microbiome modulation as a potential therapeutic treatment for intestinal pathologies and neuropsychiatric disorders involving serotonin.

## 1. Introduction

The intestinal tract is an ecosystem in which resident microbiota live in symbiosis with their host. Firmicutes and bacteroidetes are the main intestinal microbiota phyla, representing 90% of the human gut microbiota [1]. These microbes play a crucial role in maintaining immune and metabolic homeostasis. In this context, commensal bacteria maintain the integrity of the mucosal barrier, protect against pathogens, and regulate the development and function of innate and adaptive immune cells [2]. Gut microbiota also provide nutrients including vitamins and metabolize some dietary components, generating an extensive repertoire of associated by-products such as short-chain fatty acids (SCFAs) [3].

SCFAs are key metabolites with important biological functions. They are carboxylic acids, coming from the microbial fermentation of complex polysaccharides that are non-digestible by the host [4]. In humans, the highest SCFAs gut concentration is found in the colon at a molar ratio of 60:25:15 for acetate(C2):propionate(C3):butyrate(C4) [5]. Intestinal absorption of SCFAs is facilitated by passive diffusion and transport proteins as sodium-coupled monocarboxylate transporter 1/2 (SMCT1/2) and monocarboxylate transporter 1/4 (MCT1/4). The functions of SCFAs are highly vital for healthy status, as they promote lipid, glucose, and immune homeostasis [6]. SCFAs act as energy substrates for enterocytes and colonocytes, affecting the intestinal epithelial barrier and defense roles. They are involved in anti-inflammatory effects by regulating the recruitment and migration of immune cells, the differentiation of T and B cells, and the gene expression of some inflammatory chemokines and cytokines [7]. Indeed, it has been seen that butyrate and propionate are capable of reducing the activity of NF-κB and the secretion of the inflammatory factor TNFα [8]. SCFAs could also influence brain structure and function, and improve learning, memory, mood, neurodevelopment, cognitive functions, and stress responses in mammals [4]. 

Given the close relationship between the gut microbiota and the host, disbiosis is related to intestinal disorders and to several extra-intestinal diseases such as metabolic, behavioral and neurological pathologies. Microbiota manipulation and SCFAs administration have been proposed as potential therapeutic targets for such diseases [9]. 

Interestingly, microbiota and their metabolites play an essential role in the gut–brain axis through their involvement in the regulation of the serotonergic system. Serotonin (5-HT) has emerged as a leading neurotransmitter in this axis for its contribution to both GI and brain physiology, acting as a broad neuromodulator essential in the control of mood, sleep, cognition, memory, appetite, intestinal motility, nutrient absorption, and secretion of water and electrolytes [10]. Extracellular 5-HT availability is determined by serotonin transporter (SERT), which is located mainly in enterocytes, platelets, and neurons. Serotonergic signaling is due to serotonin binding to its receptors distributed broadly, especially in the gut [11]. Intestinal microbiota communicate with the innate immunity through specific receptors, such as Toll-like receptors (TLRs). In this context, microbial activation of TLR2, TLR3, TLR4, and TLR9 reduces SERT activity and levels, increases gut 5-HT extracellular concentration, and contributes to the inflammatory response, which affects gastrointestinal function [12,13]. In the same way, gut microbiota promote enteric 5-HT production through SCFAs [14], as well as phenolic and indolic compounds derived from microbes [15]. Additionally, gut bacteria can also synthesize 5-HT as well as regulate 5-HT synthesis through tryptophan metabolism [16]. 

While it is widely defined that many neuropsychiatric disorders and digestive pathologies show alterations in the serotonergic system and the microbiota, there is a knowledge gap regarding the relation of these disorders and the microbiome, i.e., through SCFAs. Therefore, in this work, our goal is to determine the impact of gut microbiota-derived SCFAs in the intestinal serotonergic system, in order to be able to find more specific targeted therapies that may be more effective and have fewer side effects.

## 2. Materials and Methods

### 2.1. Cell Culture

Caco-2/TC7 cells have been used in the present study since mimic enterocyte cells found in gastrointestinal tract have a reproducible epithelial physiology and serotonergic system machinery [17]. Cells were maintained at 37 °C and 5% CO_2_ and fed by a high-glucose Dulbecco’s modified Eagle medium (DMEM) with 2 mM glutamine, 100 U/mL penicillin, 100 μg/mL streptomycin, 1% non-essential amino acids, and 20% heat-inactivated foetal bovine serum (FBS) from Life Technologies (Carlsbad, CA, USA). The cells were passaged enzymatically with a combination of 0.25% trypsin–1 mM EDTA and sub-cultured in flasks from Sarstedt (Nuembrecht, Germany). Culture medium was changed two days after seeding and daily afterwards. The experiments were performed in cells cultured for 14 days until full enterocyte-like differentiation was achieved. Cells were treated with FBS-free culture medium containing different concentrations of acetate, propionate, and butyrate, as well as a mix of all SCFAs in the physiological proportion 60:25:15 (0.6 mM acetate, 0.125 mM propionate and 0.75 mM butyrate). Cell monolayer was analyzed before experiments, and any condition affected Caco-2/TC7 cells characteristics.

### 2.2. 5-HT Uptake Studies

Cultured cells in 24-well plates were used to perform uptake measurements, seeded at a density of 4 × 10^4^ cells/well, as previously described [18], either under a control condition and different experimental conditions. The composition of the transport medium in mM was 137 NaCl, 4.7 KCl, 1.2 KH_2_PO_4_, 1.2 MgSO_4_, 2.5 CaCl_2_, 10 HEPES pH 7.4, 4 glutamine, 1 ascorbic acid, 0.1% BSA, and both 0.2 μM 5-HT and [^3^H]-5-HT (1.5 μCi/mL) as the substrate. A 5 µL sample of this transport medium was taken as a standard measure of the radioactivity. Cells were pre-incubated at 37 °C and 5% CO_2_ prior to the experiment with 1 mL of transport medium without substrate during the last 30 min of the corresponding treatment. Subsequently, the cells were washed with 1 mL of the substrate free transport medium without any treatment substance at 37 °C, and then incubated with 0.5 mL of the transport medium at 37 °C for 6 min. The uptake was inhibited by removing the transport medium and washing the cells with 1 mL twice in a cold transport medium with 20 μM 5-HT. Then, cells were diluted in 0.5 mL of 0.1 N NaOH and a sample of 200 μL was taken for radioactivity measurement. Finally, the protein concentration was calculated by using the Pierce BCA Protein Assay kit from Thermo Fisher Scientific (Waltham, MA, USA) with BSA as the standard. To assess the radioactivity in the samples of the transport medium and the cell homogenate, 1.5 mL of scintillation liquid were added, and [^3^H] was measured (Wallac Liquid Scintillation Counter, Perkin-Elmer, Waltham, WA, USA). The measure of radioactivity is expressed in counts per minute (cpm), and all results were calculated in pmol 5-HT/mg protein and represented as a percentage of the control value (100%).

### 2.3. RNA Isolation, Reverse Transcription and Real-Time PCR

Cells were cultured in 6-well plates, seeded at a concentration of 2 × 10^5^ cells/well and total RNA was isolated using TRIzol Reagent from Sigma–Aldrich (Saint Louis, MO, USA). RNA was used as a template for cDNA synthesis using the NxGen M-MuLV Reverse transcriptase from Qiagen (GmbH, Hilden, Germany). The cDNAs were used to assess the mRNA expression by qPCR using Fast SYBR Green Master Mix from Thermo Fisher Scientific.

Quantification of SERT, 5-HTR1A, 5-HTR2A, 5-HTR2B, 5-HTR3A, 5-HTR4, and 5-HTR7 in Caco-2/TC7 cells was performed using the Step One Plus Real-Time PCR System from Applied Biosystems (Foster City, CA, USA), with GAPDH, β-actin, and HPRT1 as housekeeping genes. Primers used are shown in Table 1. Data were assessed by the Applied Biosystem Step One Software v2.3 from Applied Biosystems. The mRNA relative expression was calculated with the 2^−ΔΔCt^ method.

### 2.4. Protein Analysis by Western Blotting

Cells were cultured in 75 cm^2^ flasks at a density of 75 × 10^4^ cells/flask. Cells were washed with PBS and resuspended with an cold Tris-mannitol buffer (2 mM Tris, 50 mM Mannitol, pH 7.1) with 100 μM phenylmethylsulfonyl fluoride (PMSF), 25 μg benzamidine, a protease inhibitor cocktail (Complete Mini, EDTA-free; Roche, Barcelona, Spain), and 0.02% sodium azide. Samples were disrupted using a Potter–Elvehjem homogenizer with a PTFE pestle and the suspension was disrupted by sonication (15, 1-sbursts, 60 W). A sample was obtained from the lysate for total protein analysis and protein quantification using a Pierce BCA Protein Assay kit from Thermo Fisher Scientific, with BSA being the standard.

Cell lysate from Caco-2/TC7 cells with a concentration of total protein of 60 μg was electrophoresed in 8% SDS-PAGE gels and later transferred to PVDF membranes by electroblotting. Membranes were blocked with 4% non-fat dried milk plus 1% BSA and incubated with goat polyclonal anti-human SERT antibody 1:1000 from Abcam (Cambridge, UK). The primary antibody was detected using a secondary rabbit anti-goat Ig 1:1000 from Santa Cruz Biotechnology (Santa Cruz, CA, USA) with horseradish peroxidase and the Western Bright Sirius HRP substrate from Advansta (Menlo Park, CA, USA). In addition, the signal was visualized using VersaDoc™ from Imaging System Bio-Rad (Hercules, CA, USA). After stripping, membranes were re-incubated with goat polyclonal anti-human β-actin antibody 1:1000 from Santa Cruz Biotechnology (Santa Cruz, CA, USA) for the determination of differences in the sample loading. The SERT/β-actin protein ratio was measured in densitometry units using Quantity One 1-D Analyses Software from Bio-Rad. Data are shown as a percentage of the control values (100%).

### 2.5. Statistical Analysis

The results were illustrated as the mean ± the standard error of the mean (SEM), and differences between groups were statistically analyzed using Prism GraphPad Program (Prism version 8.01, GraphPad Software, San Diego, CA, USA). One-way analysis of variance (ANOVA) followed by Bonferroni’s (when it was needed), Kruskal–Wallis tests (non-parametric), or unpaired *t*-tests were chosen to detect differences. Previously, normal distribution was confirmed with the Kolmogorov–Smirnov test. Significance level was set to *p* < 0.05.

## 3. Results

### 3.1. Effects of SCFAs on 5-HT Uptake

**Figure 1:**
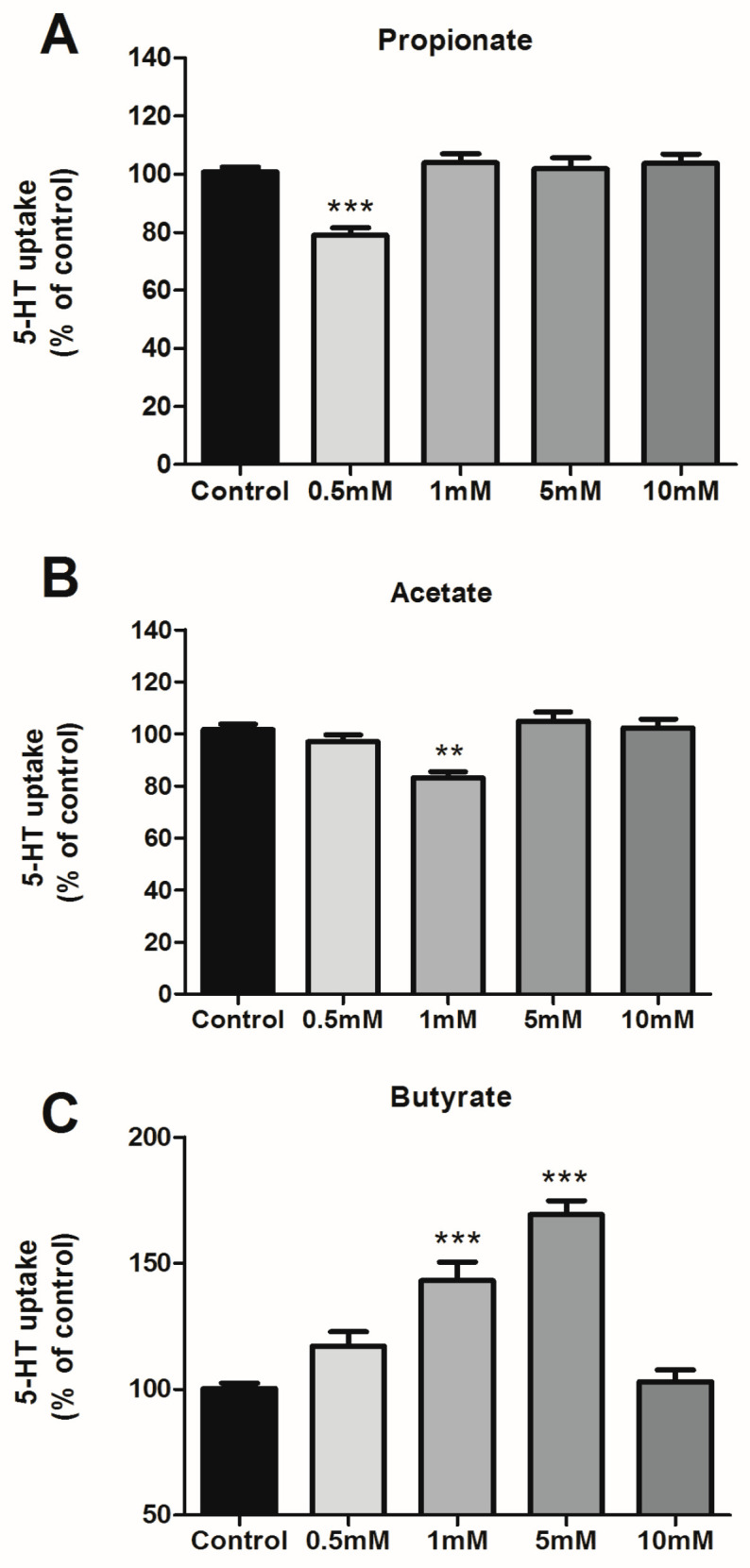
Effects of SCFAs on 5-HT uptake. 5-HT uptake was measured after a 6-min incubation of 0.2 μM 5-HT. SCFAs concentrations range from 0.5 to 10 mM during 24 h for propionate (**A**), acetate (**B**), and butyrate (**C**). Results are shown as the percentage of the control (100%) and are the mean plus SEM of four independent experiments. Absolute values were: control 6.45  ±  0.41 pmol 5-HT/mg; propionate 0.5 mM: 5.21  ±  0.63 pmol 5-HT/mg; acetate 1 mM: 5.03  ±  0.52 pmol 5-HT/mg; butyrate 1 mM: 8.08  ±  0.77 pmol 5-HT/mg; butyrate 5 mM: 7.51  ±  0.56 pmol 5-HT/mg. *** *p* < 0.001, ** *p* < 0.01 compared with the control value (untreated cells).

The effect of SCFAs on SERT activity was analyzed by 5-HT uptake in Caco-2/TC7 cells treated with propionate, acetate, and butyrate at different concentrations from 0.5 mM to 10 mM for a duration of 24 h. As shown in Figure 1A,B, propionate and acetate only reduce 5-HT uptake significantly in one specific dose (0.5 mM for propionate and 1 mM for acetate), while butyrate seems to have a dose-dependent effect from 0.5 mM to 5 mM, and no effect at 10 mM (Figure 1C). Propionate and acetate yield a significant 25% reduction of SERT activity and butyrate induces an increase of up to 50% of SERT activity.

### 3.2. SERT Expression Is Regulated by SCFAs

From previous results, we demonstrated that SCFAs affect intestinal SERT activity. To gain a more in-depth understanding of this modulation, SERT level was analyzed by measuring SERT mRNA and protein expression in cells treated with 0.5 mM propionate, 1 mM acetate, and 5 mM butyrate for one day.

**Figure 2:**
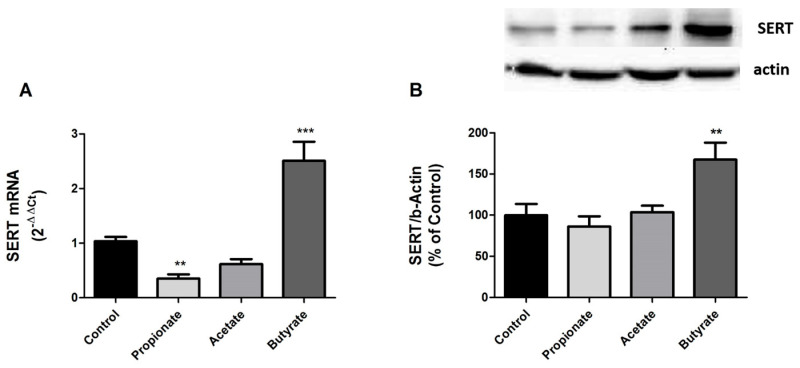
SERT expression is regulated by SCFAs. (**A**) qPCR analysis of SERT mRNA level in cells treated for 24 h with 0.5 mM propionate, 1 mM acetate, and 5 mM butyrate. Relative quantification was performed using comparative Ct method (2^–ΔΔCt^) normalized by HPRT1, GAPDH, and β-actin mean. Results are expressed as arbitrary units where the control is set to 1 and the mean ± SEM of four independent experiments. *** *p* < 0.001, and ** *p* < 0.01. (**B**) SERT protein detection by western blot in cells treated with 0.5 mM propionate, 1 mM acetate, and 5 mM butyrate for 24 h. SERT protein levels (70 kDa) were normalized with β-actin (42 kDa) as loading control (SERT/β-actin ratio). Results are shown as a percentage of the control value and are the mean ± SEM of four independent experiments. ** *p* < 0.01 compared with the control value.

Our results showed that propionate and acetate, which reduce SERT activity, also decrease SERT expression at the mRNA level, although only propionate does so significantly (Figure 2A). However, there was a slight SERT protein reduction, but it was not significant for neither of these two types of SCFAs (Figure 2B). In contrast, SERT expression at both the mRNA and protein levels was significantly increased by butyrate.

### 3.3. SCFAs Modulate the Expression of 5-HT Receptors

**Figure 3:**
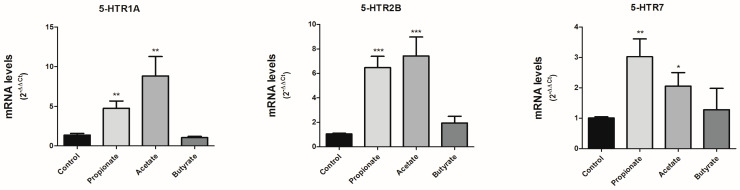
SCFAs modulate the expression of 5-HT receptors. 5-HT receptors (1A, 2B, and 7) mRNA level was assessed in cells treated for 24 h with 0.5 mM propionate, 1 mM acetate, and 5 mM butyrate by qPCR. The experiment was carried out in triplicate with the comparative Ct method (2^–ΔΔCt^) normalized by HPRT1, GAPDH, and β-actin mean. Results are expressed as arbitrary units (control  =  1) and are mean plus SEM of three independent experiments. * *p*  <  0.05, ** *p*  <  0.01 and *** *p*  <  0.001 compared with the control value.

Since SCFAs can modulate SERT expression and activity in Caco-2/TC7 cells, we explored if SCFAs could also affect other components of serotonergic system. There are no complete studies on the expression of 5-HT receptors in Caco-2/TC7 cells, so we decided to analyze the effect on the expression of the best-characterized receptors at the digestive level. 5-HTR (1A, 2A, 2B, 3A, 4, and 7) mRNA expression was assessed in our in vitro cells after treating the cells with 0.5 mM propionate, 1 mM acetate and 5 mM butyrate for one day. The expression of 5-HTR 2A, 3A and 4 was negative, suggesting that our Caco-2/TC7 cell line does not express these receptors. Interestingly, the expression of 5-HTR1A, 2B, and 7 was detected. Our findings demonstrate that propionate and acetate significantly increased the mRNA expression of 5-HTR1A, 2B and 7, whereas butyrate did not induce any alteration in the expression of these receptors (Figure 3).

### 3.4. Effects of Physiological SCFAs Mix on Serotoninergic System

The individual treatment of SCFAs, namely propionate, acetate and butyrate can regulate the intestinal serotonergic system; however, these SCFAs are produced simultaneously by gut microbiota. Therefore, we proposed to study the physiological mix of the three SCFAs on the serotonergic system. To achieve this, we treated cells with a physiological proportion of each SCFA detected in the intestinal lumen (60:25:15) [19] and assessed mRNA expression of different compounds of the serotonergic system.

**Figure 4:**
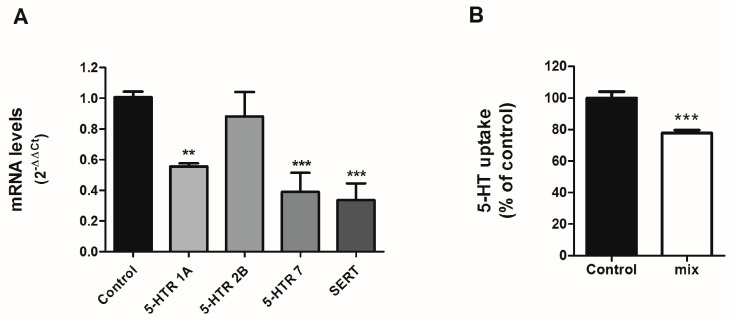
Effects on SCFAs mix on serotonergic system. Caco-2 cells were treated for 24 h with the combination (60:25:15) of 0.6 mM acetate, 0.125 mM propionate, and 0.75 mM butyrate. (**A**) qPCR determination of mRNA level. Analysis was carried out using comparative Ct method (2^–ΔΔCt^) normalized by HPRT1, GAPDH, and β-actin mean. Data are expressed as arbitrary units (control = 1) and are the mean ± SEM of four independent experiments. ** *p*  <  0.01 and *** *p*  <  0.001 compared with the control value. (**B**) 5-HT uptake measured after 6-min incubation of 0.2 μM 5-HT. The results are expressed as the percentage of the control (100%) and are the mean plus SEM of four independent experiments. Absolute control value was 6.21  ±  0.33 pmol 5-HT/mg. *** *p*  <  0.001 compared with the control value.

Our results show that the combination of the three SCFAs decreased the expression of 5-HT receptors 1A and 7, with no alteration of 5-HT receptor 2B (Figure 4A). These effects were opposite to those observed for propionate and acetate when administered individually. In addition, the combination of SCFAs resulted in a significant reduction of SERT (Figure 4A). Next, we analyzed whether this SERT reduction could impact SERT function. 5-HT uptake was measured in cells treated with the SCFA mix (0.6 mM acetate, 0.125 mM propionate, and 0.75mM butyrate) for 24 h. The combination of propionate, acetate, and butyrate not only reduced SERT mRNA, but also decreased SERT function with a 20% reduction of 5-HT uptake (Figure 4B), similar to what was observed for propionate and acetate.

## 4. Discussion

Serotonin is a key neurotransmitter linked to critical neuronal and digestive functions and plays a fundamental role as a communicator in the gut–brain axis [20]. Previous studies have described how gut microbiota can affect serotonin levels and signaling. Some microbial metabolites were found to stimulate serotonin production [21], and microbial-associated metabolites like butyric acid can stimulate enterochromaffin cells to release 5-HT [22]. Despite the widespread understanding of the involvement of microbiota-associated metabolites in serotonin host biosynthesis, little is known about how microbial metabolites like SCFAs could modulate serotonergic system. Therefore, this study focuses on delving into the potential relationship between these microbial-derived metabolites and the intestinal serotonergic system. Our results demonstrate that SCFAs can affect the intestinal serotonergic system. Propionate, acetate, and butyrate modulate SERT activity and expression and can modify the expression of 5-HT receptors. The results show that propionate and acetate behave similarly, reducing SERT activity and expression while increasing the expression of certain 5-HT receptors that intensify serotonin signaling. In contrast, butyrate enhances SERT activity and expression in a similar manner to what was observed with anti-inflammatory substances like IL-10 [23] and does not seem affect 5-HT receptors levels. Supporting our results, butyrate helps to maintain the ileal brush border structure, tight junction integrity, and protein expression while reducing intestinal inflammation responses induced by LPS in mice [24], thereby suggesting a protective role of butyrate.

Regulation of the serotonergic system is relevant for maintaining intestinal homeostasis. Abnormal changes in SERT and 5-HT receptors affects 5-HT signaling and can lead to development of gut functional disorders [25,26] including inflammatory bowel disease (IBD). These results would suggest that butyrate has anti-inflammatory potential by helping to increase the uptake of 5-HT, reducing the bioavailability of 5-HT, and therefore, reducing the pro-inflammatory effects of 5-HT. This could be an interesting finding considering that butyrate is one of the preferred metabolic substrates for some intestinal cells, such as colonic epithelial cells [27]. Studies have reported that IBD patients show a decrease in the abundance of butyrate-producing bacteria and butyrate content. In fact, this study shows a reduction in colonocyte butyrate oxidation, lower luminal anaerobiosis, and a facilitation in the expansion of Enterobacteriaceae that contribute to inflammation [28]. Moreover, faecal acetate and propionate levels are markedly decreased in patients with IBD compared with healthy individuals [29]. Similar to butyrate, this could support their anti-inflammatory effect, contrasting with the down-regulation observed in SERT activity and expression induced by acetate and propionate. However, this discrepancy may suggest a hypothetical reduction in acetate and propionate levels in IBD patients as a potential compensatory mechanism to reverse the inflammation generated by these SCFAs, thereby justifying the results of the present study. Furthermore, SCFAs could not only regulate SERT activity, but also stimulate 5-HT production. Previous studies have determined that gut microbiome promotes 5-HT production by enhancing TPH1, the major 5-HT synthesis enzyme in enterochromaffin cells [14].

Similarly, it has been recognized that other bacterial metabolites can also modulate intestinal serotonergic system. In this context, supernatant derived from *L. reuteri* upregulates SERT expression in human colonic T84 cells [30] and L-lactate, another bacterial metabolite, increase the expression of the regulatory protein P11, which in turn controls and increases the expression of 5-HTR1B, 5-HTR1D, and 5-HTR4 on the surface of central nervous cells [31].

Our study demonstrated that SCFAs not only modulate SERT, but can also regulate 5-HT receptors expression. While butyrate does not appear to alter receptor expression, propionate and acetate significantly increased the mRNA expression of 5-HTR1A, 2B, and 7. 5-HTR1A, which is expressed in intestinal epithelium and enteric neurons. Its activation causes 5-HT release from enterochromafin cells [17]. Therefore, the augmentation of this receptor induced by propionate and acetate would contribute the increase of 5-HT signaling. Similarly, 5-HTR2B seems to also increase 5-HT signaling, as it blocks SERT activity, thereby increasing 5-HT extracellular levels [32]. Finally, 5-HTR7 plays a critical role in smooth muscle relaxation in the gut. High levels of 5-HT7 receptor-expressing mucosal nerve fibers were observed in the colon of patients with irritable bowel syndrome (IBS), which is characterized by motility dysfunctions. In addition, 5-HTR7 would be involved in intestinal hyperalgesia [33].

At the level of physiological condition, the three SCFAs are produced simultaneously in a constant proportion. Therefore, we have also assessed the effect of SCFAs combination. The three SCFAs mix reduced the expression of 5-HT receptors 1A and 7 with no alteration of 5-HT receptor 2B. The effects on 5-HT receptors induced by the combined treatment can be controversial, as in the case of 5-HT receptor 2B. This receptor is not modified with combined treatment, despite single treatment with acetate or propionate increasing their expression. Butyrate does not alter 2B receptor expression. This can be explained by the fact that the intracellular pathways activated by SCFAs may be the same, which neutralizes their effects. Furthermore, the combination of SCFAs yields a decrease of SERT expression, and this reduction is also followed by a significant reduction in 5-HT uptake. Although SCFAs mix induced a 20% reduction of SERT activity, the reduction was not additive between the three SCFAs, indicating that the intracellular pathways used by SCFAs could be the same. The activation of several pathways by SCFAs such as cAMP-PKA [34], ERK1/2 [35], and p38 MAPK [36] has been described, and all these intracellular pathways have been previously linked to SERT modulation [37]. In this context, we have observed that low concentrations of acetate, propionate, and butyrate are able to modify SERT activity, while higher concentrations did not alter SERT function. High concentrations of SCFAs may block the expected down-regulation of SERT directly by acting on the protein allosteric sites or indirectly by activating intracellular pathways that maintain a similar 5-HT uptake. This may be a typical effect, as observed with other important substances, such as adenosine [38]. In this sense, SCFAs could be a homeostatic gut component that ensures a low pro-inflammatory level while participating in the intestinal defense against external aggressions.

Several studies have described the important impact of intestinal serotonergic alterations on inflammation development and severity within the gut. For example, IBD patients suffer from dysregulation of 5-HT synthesis and alterations in 5-HTR expression, which contribute to IBD-associated symptoms such as abnormal gut motility and sensations of pain. An increased number of enterochromaffin cells and a reduced SERT expression have also been observed in these pathologies with an increase of extracellular serotonin availability in the lumen and in the gut [24]. In this context, intestinal serotonergic system could be modulated by microbiota as another way of communication between gut microbiota and the body.

In summary, we described another pathway through which commensal microbes can modulate 5-HT signaling in the host. Our results show that SCFAs can regulate the serotonergic system by the modulation of SERT and specific 5-HT receptors. Given the importance of serotonin in host physiology, we suggest that the regulation of SERT and 5-HT receptors by SCFAs are essential in both GI and central nervous system homeostasis. Metabolites synthesized by gut microbes from dietary source are linked to key features in neuronal and digestive processes and are associated to dysfunction of the gut–brain axis. Modulating microbial metabolites through dietary changes [39] or targeting gut microbiota could be an interesting therapeutic strategy for the prevention and treatment of intestinal disorders [40], as well as non-intestinal pathologies such as neurological and neuropsychiatric diseases [41]. For example, some recent investigations have described changes in faecal SCFAs levels following faecal microbiota transplantation as a therapeutic treatment for IBS patients [42], suggesting that direct and non-direct SCFAs modulation could be an effective therapeutic target for inflammatory pathologies.

A better understanding of the action mechanism of SCFAs in the serotonergic system will facilitate potential applications of these microbial metabolites in 5-HT-related diseases. We suggest that future studies should determine the effects of SCFAs at in vivo level. There is no doubt about the great potential therapeutic tool of microbiota manipulation for the treatment of many diseases, such as the ones including in the gut–brain axis disorders.

## Figures and Tables

**Table 1:** Primer sequences used for qPCR.

Name	Forward Primer (5′-3′)	Reverse Primer (5′-3′)
SERT	AAATCCAAGCACCCAGAGAT	AGACTGTGTCCCTGTGGAGA
5-HTR_1A_	AACAACAACACATCACCACCGGC	AGATGCTCCATGGCGGGTGT
5-HTR_2A_	CAACTACGAACTCCCTAATG	AAACAGGAAGAAGACGATGC
5-HTR_2B_	GAATCACAGAAAACAGCAAATGG	CCCATTTCGAATTCCATGTT
5-HTR_3A_	GCCCTACTTTCGGGAGTTCAGCAG	TCTTGGTGGCTTGGGAGGTG
5-HTR_4_	CCTGTAATGGACAACTTGA	CCATGTTATTCCAGCCTTG
5-HTR_7_	AGAGAAGCCAGACGGAGAGAA	TACGGCAGAGTCGAGAAAGTG
GAPDH	CATGACCACAGTCCATGCCATCACT	TGAGGTCCACCACCCTGTTGCTGTA
HPRT1	CTGACCTGCTGGATTACA	GCGACCTTGACCATCTTT
β-actin	AGCACGGCATCGTCACCAACT	ACATGGCTGGGGGTGTTGAAAGG

## Data Availability

Data are available upon reasonable request to the corresponding author.
